# Fucosyllactose and L-fucose utilization of infant *Bifidobacterium longum* and *Bifidobacterium kashiwanohense*

**DOI:** 10.1186/s12866-016-0867-4

**Published:** 2016-10-26

**Authors:** Vera Bunesova, Christophe Lacroix, Clarissa Schwab

**Affiliations:** 1 Laboratory of Food Biotechnology, ETH Zurich, Institute of Food, Nutrition and Health, Schmelzbergstrasse 7, Zurich, Switzerland; 2 Department of Microbiology, Nutrition and Dietetics, Faculty of Agrobiology, Food and Natural Resources, Czech University of Life Sciences, Prague, Czech Republic

**Keywords:** Bifidobacterium, HMOs, fucosyllactose, L-fucose, 1,2 propanediol

## Abstract

**Background:**

Human milk oligosaccharides (HMOs) are one of the major glycan source of the infant gut microbiota. The two species that predominate the infant bifidobacteria community, *Bifidobacterium longum* subsp. *infantis* and *Bifidobacterium bifidum,* possess an arsenal of enzymes including α-fucosidases, sialidases, and β-galactosidases to metabolise HMOs. Recently bifidobacteria were obtained from the stool of six month old Kenyan infants including species such as *Bifidobacterium kashiwanohense,* and *Bifidobacterium pseudolongum* that are not frequently isolated from infant stool.

The aim of this study was to characterize HMOs utilization by these isolates. Strains were grown in presence of 2′-fucosyllactose (2′-FL), 3′-fucosyllactose (3′-FL), 3′-sialyl-lactose (3′-SL), 6′-sialyl-lactose (6′-SL), and Lacto-N-neotetraose (LNnT). We further investigated metabolites formed during L-fucose and fucosyllactose utilization, and aimed to identify genes and pathways involved through genome comparison.

**Results:**

*Bifidobacterium longum* subsp. *infantis* isolates*, Bifidobacterium longum* subsp. *suis* BSM11-5 and *B. kashiwanohense* strains grew in the presence of 2′-FL and 3′- FL. All *B. longum* isolates utilized the L-fucose moiety, while *B. kashiwanohense* accumulated L-fucose in the supernatant. 1,2-propanediol (1,2-PD) was the major metabolite from L-fucose fermentation, and was formed in equimolar amounts by *B. longum* isolates.

Alpha-fucosidases were detected in all strains that degraded fucosyllactose. *B. longum* subsp. *infantis* TPY11-2 harboured four α-fucosidases with 95–99 % similarity to the type strain. *B. kashiwanohense* DSM 21854 and PV20-2 possessed three and one α-fucosidase, respectively. The two α-fucosidases of *B. longum* subsp. *suis* were 78–80 % similar to *B. longum* subsp. *infantis* and were highly similar to *B. kashiwanohense* α-fucosidases (95–99 %). The genomes of *B. longum* strains that were capable of utilizing L-fucose harboured two gene regions that encoded enzymes predicted to metabolize L-fucose to L-lactaldehyde, the precursor of 1,2-PD, via non-phosphorylated intermediates.

**Conclusion:**

Here we observed that the ability to utilize fucosyllactose is a trait of various bifidobacteria species. For the first time, strains of *B. longum* subsp. *infantis* and an isolate of *B. longum* subsp. *suis* were shown to use L-fucose to form 1,2-PD. As 1,2-PD is a precursor for intestinal propionate formation, bifidobacterial L-fucose utilization may impact intestinal short chain fatty acid balance. A L-fucose utilization pathway for bifidobacteria is suggested.

**Electronic supplementary material:**

The online version of this article (doi:10.1186/s12866-016-0867-4) contains supplementary material, which is available to authorized users.

## Background

Bifidobacteria are universally distributed in organisms that raise offspring by parental care including mammals, birds and social insects. Bifidobacteria are highly specialized organisms in using non-digestible oligosaccharides and a major part of their genomes is devoted to the utilization of carbon sources [1–5]. The proportion of genes related to carbohydrate transport and metabolism is higher in bifidobacteria than in *Bacteroides,* which are also characterized by their ability to utilize a variety of polysaccharides [6]. Host-specific adaption in regard to carbohydrate degradation has been suggested [7, 8]. Adult species, such as *Bifidobacterium adolescentis* and *Bifidobacterium longum* subsp. *longum*, are well equipped to degrade plant derived polysaccharides [4, 9]. Infant species, such as *Bifidobacterium longum* subsp. *infantis* and *Bifidobacterium bifidum*, are adapted to utilize human milk oligosaccharides (HMOs), one of the major glycan sources of breast milk [3, 10–12]. Primary components of HMOs are D-glucose, D-galactose, L-fucose, N-acetylglucosamine, and sialic acid. Lactose constitutes the reducing end, its galactose moiety can be fucosylated or sialylated to form 2′- or 3′-fucosyllactose (2′-FL or 3′-FL) or 3′- and 6′-sialyl-lactose (3′-SL or 6′-SL). Lactose can also be elongated with units of N-acetyllactosamine (Gal-β1-4GlcNAc) with its simplest form being Lacto-N-neotetraose (LNnT) [13].

The degradation of HMOs relies on a complex network of extracellular solute binding proteins, transporters and intra- or extracellular glycosyl hydrolases (GH). Both *B. longum* subsp. *infantis* and *B. bifidum* harbour several α-fucosidase and sialidases, hexosaminidases, lacto-N-biosidases, α- and β-galactosidases to degrade HMOs. *B. longum* subsp. *infantis* degrades HMOs intracellularly, while *B. bifidum* harvests HMOs extracellularly through the activity of membrane bound enzymes [3, 12]*.* Other species recovered from infant stool, such as *Bifidobacterium breve*, have a limited capacity to degrade HMOs, however, they can profit from cross-feeding of HMO constituents released by *B. bifidum* [14–16].

Bifidobacteria metabolize hexoses via the ‘bifid shunt’ with fructose-6-phosphoketolase being the key enzyme to theoretically yield 1.5 mol acetate, 1 mol lactate and 2.5 ATP from 1 mol glucose [17]. The ratios of lactate and acetate formed may vary with carbohydrate source and species, depending on whether the intermediate pyruvate is cleaved to acetyl phosphate and formate, or reduced to lactate [18]. Pentoses, such as xylose, are also fermented to lactate, acetate and possible formate [19]. There is little information available about bifidobacterial metabolism of desoxyhexoses, and rhamnose was not used by various species tested [6].

In a previous study, several *Bifidobacterium* strains were isolated from Kenyan infant stool, that were identified as *B. longum*, *B. bifidum*, *B. breve*, *Bifidobacterium kashiwanohense,* and *Bifidobacterium pseudolongum* [20]*. B. kashiwanohense* has only been isolated from a healthy Japanese infant [21] and from Kenyan anaemic infants [20]. *B. pseudolongum* has been frequently recovered from animal feces [22]. *B. kashiwanohense,* and *B. pseudolongum* are species not commonly associated with the infant bifidobacteria community, however, the presence of additional species in infant feces from developing countries, might reflect variations in diet and sanitary status. Little is known about the ability of these species to utilize HMOs.

Therefore it was the aim of this study to investigate HMO degradation by these newly obtained isolates. As we observed that beside *B. longum* subsp. *infantis* and *B. bifidum,* an isolate of *B. longum* subsp. *suis*, and strains of *B. kashiwanohense* were able to metabolise fucosyllactose, we further investigated metabolite formation during growth on L-fucose and fucosyllactose. Furthermore we analyzed genomes of the studied strains to elucidate possible genes and pathways involved in fucosyllactose degradation and L-fucose utilization through genome comparison.

## Methods

### Bacterial strains

Twenty-nine bifidobacterial strains were included in the initial HMO utilization screening (Table 1). Nineteen strains originated from stool samples of Kenyan infants [20] and ten reference strains were obtained from the Deutsche Sammlung von Mikroorganismen und Zellkulturen GmbH (DSMZ, Braunschweig, Germany, Table 1). Kenyan isolates that had been previously typed to species level were additionally characterized on subspecies level using the (partial) 16S rRNA gene as marker. Briefly, DNA was extracted from overnight cultures using the PrepMan® Ultra protocol for pure culture (Thermo Fisher Scientific, Reinach, Switzerland). PCR amplification of partial 16S rRNA genes was performed using universal primers 518 F (5′-CCAGCAGCCGCGGTAATACG–3′) and 1391R (5′– GACGGGCGGTGTGTRCA–3′). PCR reaction mixtures (25 μL) contained 12.5 μL of 2× PCR MasterMix (Thermo Fisher Scientific), 0.2 μM of primers (Microsynth AG, Balgach, Switzerland) and 1 μl of template DNA. The cycling programme consisted of an initial denaturation of 5 min at 95 °C, followed by 32 cycles of denaturation for 30 s at 95 °C, annealing for 30 s at 52 °C, and extension for 1 min at 72 °C. Amplicons were sequenced by GATC Biotech (Konstanz, Germany).

**Table 1:** Strains used for HMO utilization screening

Species	Strain code	Origin
*B. bifidum*	DSM 20456	stool of breast-fed infant
*B. bifidum*	BRS26-2	Kenyan infant stool, 6 m old
*B. bifidum*	BSM2-3	Kenyan infant stool, 6 m old
*B. bifidum*	BRS-300	Kenyan infant stool, 6 m old
*B. bifidum*	BRS27-3	Kenyan infant stool, 6 m old
*B. bifidum*	BSM28-1	Kenyan infant stool, 6 m old
*B. bifidum*	TPY6-2	Kenyan infant stool, 6 m old
*B. bifidum*	DSM 20082	intestine of adult
*B. bifidum*	DSM 20215	intestine of adult
*B. bifidum*	DSM 20239	stool of breast-fed infant
*B. breve*	DSM 20213	intestine of infant
*B. breve*	TPY10-1	Kenyan infant stool, 6 m old
*B. breve*	TPY5-1	Kenyan infant stool, 6 m old
*B. kashiwanohense*	DSM 21854	Japanese infant stool, 1.5 y old
*B. kashiwanohense*	PV20-2	Kenyan infant stool, 6 m old
*B. kashiwanohense*	TPY11-1	Kenyan infant stool, 6 m old
*B. kashiwanohense*	BSM11-1	Kenyan infant stool, 6 m old
*B. longum* subsp. *infantis*	DSM 20088	intestine of infant
*B. longum* subsp. *infantis*	BRS8-2	Kenyan infant stool, 6 m old
*B. longum* subsp. *infantis*	TPY12-1	Kenyan infant stool, 6 m old
*B. longum* subsp. *infantis*	BRS8-1	Kenyan infant stool, 6 m old
*B. longum* subsp. *infantis*	TPY8-1	Kenyan infant stool, 6 m old
*B. longum* subsp. *infantis*	BSM12-2×	Kenyan infant stool, 6 m old
*B. longum* subsp. *longum*	DSM 20219	intestine of adult
*B. longum* subsp. *suis*	BSM11-5	Kenyan infant stool, 6 m old
*B. pseudolongum* subsp. *pseudolongum*	DSM 20099	pig faeces
*B. pseudolongum* subsp. *globosum*	DSM 20092	Rumen
*B. pseudolongum* subsp. *globosum*	PV8-2	Kenyan infant stool, 6 m old
*B. pseudolongum* subsp. *globosum*	BSM8-1	Kenyan infant stool, 6 m old

For generation of phylogenetic trees, 16S rRNA gene sequences were aligned and cut using CLUSTALW implemented in BioEdit Version 7. Phylogenetic analysis of partial 16S rRNA gene sequences (772 bp) was performed using Maximum Likelihood Analysis implemented in MEGA6 [23], applying the Jones-Taylor-Thornton substitution model and default settings. Bootstrap support was calculated for 500 replicates, strains of *Lactobacillus* were applied as outgroup. Sequences are listed in the Additional file 1.

Based on partial 16S rRNA gene sequences, the Kenyan isolates were characterized as *B. bifidum* (*n* = 6), *B. breve* (*n* = 2), *Bifidobacterium kashiwanohense* (*n* = 3), *Bifidobacterium pseudolongum* subsp. *globosum* (*n* = 2), *B. longum* subsp. *infantis* (*n* = 5) and *B. longum* subsp. *suis* (*n* = 1) (Table 1).

### Growth conditions

*Bifidobacterium* strains were routinely cultured at 37 °C in Wilkins-Chalgren Anaerobe Broth (Oxoid, Basel, Switzerland) supplemented with soya peptone (5 g L^−1^, Oxoid), Tween 80 (1 mL L^−1^, Sigma-Aldrich, Buchs, Switzerland), and fresh sterile filtered l-cysteine hydrochloride (0.5 g L^−1^, Sigma-Aldrich). Carbohydrate utilization profile of bifidobacteria was investigated in API 50CHL Medium (10 g L^−1^ bovine/porcine origin polypeptone, 5 g L^−1^ yeast extract, 1 mL^−1^ Tween 80, 2 g L^−1^ dipotassium phosphate, 5 g L^−1^ sodium acetate, 2 g L^−1^ di-ammonium citrate, 0.2 g L^−1^ magnesium sulphate heptahydrate, 0.05 g L^−1^ manganese sulphate monohydrate, 0.17 g L^−1^ bromocresol purple; BioMérieux, Genève, Switzerland). The pH of the API medium was adjusted to 7.5 to obtain a final pH of 7 after autoclaving at 121 °C for 15 min. Carbohydrates (concentration as indicated) were filter sterilized and added after autoclaving. Fresh sterile filtered l-cysteine hydrochloride was always added before cultivation (0.5 g L^−1^). Glucose, lactose, and L-fucose were obtained from Sigma-Aldrich, 2′-fucosyllactose (2′-FL, Fucα1-2Galβ1-4Glc), 3′-fucosyllactose (3′-FL, Fucα1-3Galβ1-4Glc), 3′-sialyl-lactose (3′-SL, NeuAcα2-3Galβ1-4Glc), 6′-sialyl-lactose (6′-SL, NeuAcα2-6Galβ1-4Glc), Lacto-N-neotetraose LNnT (Galβ1-4GlcNacβ1-3Galβ1-4Glc) were donated by Glycom A/S (Lyngby, Denmark).

### Utilization of selected sugars and metabolite formation

Isolates derived from −80 °C stock cultures were streaked on supplemented Wilkins-Chalgren agar and were incubated anaerobically at 37 °C for two days. Single colonies of each isolate were subsequently incubated twice in supplemented Wilkins-Chalgren broth (10 ml, 1:10) at 37 °C for 20 h. To obtain working cultures, the supernatant was removed from overnight cultures, cells were washed, and re-suspended in same volume of 50 mM phosphate buffer, pH 6.5 (PB).

The initial HMO utilization screening was conducted in 96-well microtiter plates. Cell suspensions (20 μl) were added to 180 μl carbohydrate supplemented API medium (2′-FL and 3′-FL: 4 mM, 3′-SL and 6′-SL: 2 mM, LNnt: 1 mM; glucose: 6 mM; lactose: 3 mM). HMOs were also alone or supplied together (HMO mixture). Glucose and lactose were used to verify suitability of the assay. Strains were grown in independent triplicates under anaerobic condition (GENbag anaer; BioMérieux, Genève, Switzerland) at 37 °C for 48 h.

To investigate growth on fucosyllactose and L-fucose of selected strains, cell suspensions (50 μl) were added to 950 μl API medium supplied with 30 mM L-fucose, 2′-FL, or 3′-FL (28.0 and 27.0 mM, respectively). Strains were grown in independent triplicates under anaerobic condition at 37 °C for 48 h.

### L-fucose utilization and metabolite analysis using high performance liquid chromatography with refractive index detection (HPLC-RI)

L-fucose, lactate, acetate and 1,2-PD concentrations were determined using HPLC (Merck-Hitachi, Darmstadt, Germany) equipped with an Aminex HPX-87H column (300 × 7.8 mm; BioRad, Cressier, Switzerland) and a RI detector. Samples were centrifuged at 13 000 *g* for 5 min at 4 °C. Supernatants (40 μL injection volume) were eluted with 10 mM H_2_SO_4_ at a flow rate of 0.6 ml min^−1^ at 40 °C. L-fucose, lactate, acetate, and 1,2-PD were quantified using external standards.

### Analysis of HMO degradation using high performance anion exchange chromatography with pulsed amperometric detection (HPAEC-PAD)

HMO degradation was investigated using HPAEC-PAD, on Dionex IC3000 equipped with a CarbopacPA20 column (Thermo Fisher Scientific, Reinach, Switzerland) and an electrochemical detector with a gold electrode. Water (A), 200 mM NaOH (B), and 1 M Na-acetate (C) were used as solvents at a flow rate of 0.25 mL min^−1^. For HMO separation, a gradient of: 0 min 30.4 % B, 1.3 % C, 22 min 30.4 % B, and 11.34 % C followed by washing and regeneration was applied. HMOs and L-fucose were identified using external standards.

### Genome sequencing

DNA was isolated from overnight culture of *B. longum* subsp. *infantis* TPY12-1 and *B. longum* subsp. *suis* BSM11-5 using the FastDNA SPIN Kit for Soil (MP Biomedicals, France) including a bead-beating procedure for cell disruption. Genome libraries of *B. longum* subsp. *infantis* TPY12-1 were sequenced with an Illumina HiSeq 2500 to obtain paired-end reads of 2x100 bp. Genome libraries of *B. longum* subsp. *suis* BSM11-5 were sequenced with an Illumina MiSeq to obtain paired-end reads of 2x150 bp. Sequencing was conducted at the Functional Genomic Center Zürich (FGCZ, Zürich, Switzerland).

### Genome assembly and annotation

Genomes were assembled using Abyss v.1.9.0 for paired-end libraries implemented in Bio-Linux 8. The partial genomes were functionally annotated with RAST using default settings [24]. RAST annotations of genes of interest were verified using the BLAST tool implemented in RAST. Average nucleotide identity (ANI) was calculated using the online tool supplied by Rodriguez-R and Konstantinidis [25]. Carbohydrate-active enzymes were selectively confirmed based on similarity to the carbohydrate active enzyme (CAZy) database entries, and Pfam alignments implemented at the CAZymes Analysis Toolkit (CAT) [26]. Additionally, dbCAN was used for identification of carbohydrate active proteins which is based on a search for signature domains of every CAZyme family [27].

## Results and discussion

### Utilization of HMOs

We investigated growth of 19 bifidobacterial isolates of Kenyan infants and 10 culture collection strains (Table 1) in the presence of individual HMOs: 2′-FL, 3′-FL, 3′-SL, 6′-SL, and LNnT, and combined HMOs in API medium. All isolates were able to grow in the presence of glucose or lactose confirming the suitability of the assay (Table 2). Growth correlated with the degradation of the supplied HMOs as determined with HPAEC-PAD for selected strain-HMO combinations (Table 2).

**Table 2:**
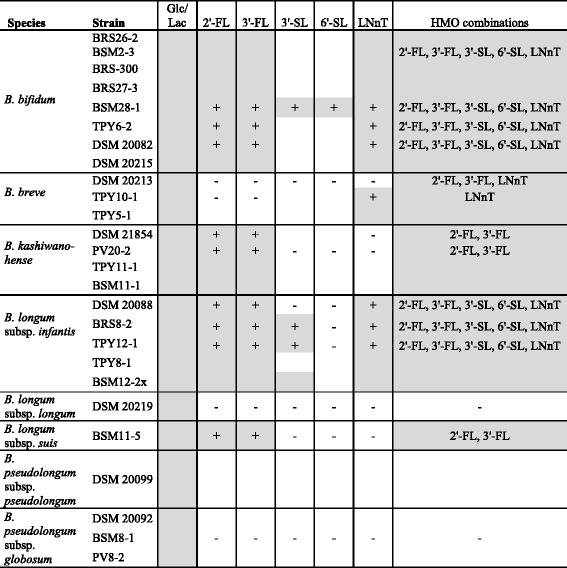
Degradation of HMOs by selected strains

Growth is indicated by grey shading. Degradation of HMOs of selected samples was investigated by HPAEC-PAD

Plus (+) indicates degradation of HMO tested, minus (−) no degradation

HMOs that were used during growth in the presence of HMO combinations (2′-FL, 3′-FL, 3′-SL, 6′-SL, LNnT) are indicated in the respective column

**Fig. 1:**
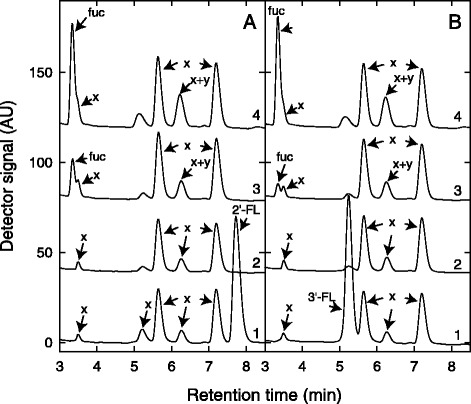
Degradation of 2′-FL (**a**) and 3′-FL (**b**) and accumulation of L-fucose. Shown are (1) unfermented control, (2) *B. longum* subsp. *infantis* DSM 20088, (3) *B. kashiwanohense* PV20-2, and (4) *B. bifidum* BSM28-1 as representatives of *B. longum, B. kashiwanohense* and *B. bifidum* isolates investigated. x, undefined media components; y, intermediate degradation compound of fucosyllactose metabolism; fuc, L-fucose

*B. longum* subsp. *infantis* utilized of 2′-FL, 3′-FL, 3′-SL and LNnT and degraded all HMOs when supplied together. *B. bifidum* grew in the presence of 2′-FL, 3′-FL and LNnT and also utilized 3′-SL and 6′-SL in HMO mixtures confirming adaptation of both species to HMO utilization, as reported before [3, 12]. Strains of *B. bifidum* liberated L-fucose and a second degradation product (Fig. 1, peak y) in the supernatant when grown in the presence of fucosyllactose while L-fucose accumulation or the release of any other degradation intermediate was not observed for *B. longum* subsp. *infantis* strains [28] (Fig. 1).

All *B. breve* isolates were able to utilize LNnT as shown previously [15]. *B. breve* DSM 20213 also degraded 2′-FL and 3′-FL when grown with HMO mixtures.

*B. longum* subsp. *suis* has not been shown to utilize of HMOs [11]. Here we identified an isolate *B. longum* subsp. s*uis* BSM11-5 able to metabolize 3′-FL and 2′-FL (Fig. 1). L-fucose was not accumulated when *B. longum* subsp. s*uis* BSM11-5 was grown with 4 mM fucosyllactose.

Also, *B. kashiwanohense* DSM 21854 and the Kenyan isolates grew in the presence of 2′-FL and 3′-FL, thereby accumulating L-fucose and releasing compound y (Fig. 1). The amount of L-fucose released by *B. kashiwanohense* isolates was only about 12 % compared to the complete release of *B. bifidum.*

Strains of *B. pseudolongum* did not metabolize with any of the HMOs tested.

The ability to use fucosyllactose was thus identified as being a trait of several bifidobacteria species. *B. longum* subsp. *suis* and *B. kashiwanohense* have not considered infant bifidobacteria species, yet, the ability to utilize fucosyllactose points at adaptation to the infant gut.

### L-fucose metabolism of bifidobacteria

Similar to *B. bifidum*, *B. kashiwanohense* excreted L-fucose into the supernatant [28]. L-fucose accumulation was not observed when *B. longum* subsp. *infantis* isolates and *B. longum* subsp. *suis* BSM 11–5 were grown in the presence of fucosyllactose.

Several clostridia and *E. coli* are capable of metabolizing L-fucose to 1,2 propanediol (1,2-PD) [29–33]. To investigate whether bifidobacteria are able to form 1,2-PD from L-fucose, *B. longum* subsp. *infantis* DSM 20088 and TPY12-1, *B. longum* subsp. *suis* BSM11-5*,* and *B. kashiwanohense* DSM 21854 were cultivated in API medium supplied with 30 mM L-fucose. As growth with L-fucose as sole carbohydrate source was unreliable, trace amounts of glucose (0.4 mM) were added to enable initial growth. When glucose was present, L-fucose was partially used and equimolar amounts of 1,2-PD were formed by the two strains of *B. longum* subsp. *infantis,* and by *B. longum* subsp. *suis* BSM11-5 (Table 3). *B. longum* subsp. *suis* BSM11-5 also formed equimolar amounts of acetate (Table 3). In contrast *B. kashiwanohense* DSM 21854 did not grow with L-fucose and trace amounts of glucose.

**Table 3:** L-fucose utilization and metabolite formation

Strain	Substrate used	Metabolite formed		
	L-fucose (mM)	lactate (mM)	acetate (mM)	1,2-PD (mM)
*B. longum* subsp. *infantis* DSM 20088	−3.4 ± 0.3	4.0 ± 0.5	1.3 ± 1.3	3.9 ± 0.8
*B. longum* subsp. *infantis* TPY12-1	−6.2 ± 0.8	3.7 ± 3.8	1.6 ± 2.2	5.3 ± 0.4
*B. longum* subsp. *suis* BSM11-5	−11.4 ± 2.9	5.6 ± 2.2	10.5 ± 2.7	10.0 ± 3.1
*B. kashiwanohense* DSM 21854	0	4.0 ± 1.7	3.5 ± 1.4	0

Strains were grown in API medium with L-fucose (30 mM) and 0.4 mM glucose as carbohydrate sources for 48 h. L-fucose, lactate, acetate, and 1,2-PD were analysed with HPLC-RI (*n* = 3)

We also investigated whether 1,2-PD was formed from 3′-FL and 2′-FL (27 and 28 mM, respectively) (Table 4). In the presence of 2′-FL and 3′-FL, *B. longum* subsp. *infantis* DSM 20088 produced a lactate:acetate ratio of 2:3 as expected of the metabolism of hexoses through the bifid shunt [17] in addition, this strain produced 1,2-PD (Table 4).

**Table 4:** Fucosyllactose utilization, metabolite formation and L-fucose accumulation

Strain	Substrate		Metabolite formed			
	Supplied	used (mM)	L-fucose (mM)	lactate (mM)	acetate (mM)	1,2-PD (mM)
*B. longum* subsp. *infantis* DSM 20088	2′-FL	−24.0 ± 4.4	0.7 ± 1.1	18.0 ± 2.7	29.0 ± 6.6	6.8 ± 1.5
	3′-FL	−23.6 ± 0.2	0	14.0 ± 0.8	23.5 ± 0.7	7.1 ± 0.2
*B. longum* subsp. *suis* BSM11-5	2′-FL	−20.1 ± 4.1	5.2 ± 0.7	13.1 ± 4.4	13.3 ± 4.8	3.2 ± 0.4
	3′-FL	−24.7 ± 3.9	0.5 ± 0.5	10.3 ± 1.3	27.3 ± 7.0	7.5 ± 1.2
*B. kashiwanohense* DSM 21854	2′-FL	−25.0 ± 0.2	10.5 ± 0.2	12.3 ± 1.0	18.2 ± 1.3	0
	3′-FL	−19.9 ± 2.1	10.2 ± 0.1	11.3 ± 0.8	19.2 ± 1.1	0

Strains were grown with 3′-FL or 2′-FL (28 and 27 mM. respectively) as sole carbohydrate source for 48 h. 2′-FL and 3′-FL concentrations were determined with HPAEC-PAD, L-fucose, lactate, acetate, and 1,2-PD were analysed with HPLC-RI (*n* = 3)

In contrast, the ratio of lactate:acetate of *B. longum* subsp. *suis* BSM 11–5 grown with 2′-FL and 3′-FL was approx. 1:1 and 1:3 respectively. *B. longum* subsp. *suis* BSM 11–5 synthesized 1,2-PD mainly from 3′-FL, and accumulated 5 mM L-fucose when grown in the presence of 2′-FL. L-fucose might have been accumulated during growth in the presence of 28 mM 2′-FL as glucose and galactose became also available after fucosyllactose degradation.

*B. kashiwanohense* DSM 21854 grew in the presence of 2′-FL and 3′FL and accumulated approximately 10 mM L-fucose but did not produce any 1,2-PD (Table 4). The ratio of lactate:acetate was approx. 1:2.

*B. longum* subsp. *infantis* degrades HMOs internally [3, 34]. The gap in substrate consumption, L-fucose release and/or 1,2-PD formation observed for *B. longum* subsp. *infantis* and *B. longum* subsp. *suis* might be due to the intracellular which were not released in the supernatant. In contrast, fucose and an additional compound were detected in supernatants of *B. bifidum* which harvests fucosyllactose extracellularly [28, 34].

We here identified 1,2-PD as a metabolite of bifidobacteria fucosyllactose respective L-fucose degradation. L-fucose derived 1,2-PD can be further metabolized to propionate and propanol by other gut microbes such as *Eubacterium hallii* [35, 36]. It was estimated that in adults approximately 30 % of propionate might derive from 1,2-PD, but no data exists for infants [36, 37]. Nevertheless, the bifidobacterial formation of lactate and 1,2-PD as precursors of short chain fatty acids butyrate and propionate, respectively, contributes to the trophic interactions of the infant gut microbiota [38].

### Genome comparison

Strains of *B. longum* subsp. *infantis*, and *B. longum* subsp. *suis* used the L-fucose moiety of 2′-FL and 3′-FL to form 1,2-PD, but the enzymatic pathways of bifidobacterial L-fucose metabolism are not known [3]. To further elucidate putative fucosyllactose and L-fucose utilization pathways, we generated draft genomes of *B. longum* subsp. *infantis* TPY12-1 and *B. longum* subsp. *suis* BSM11-5 for comparing genome data with type strain *B. longum* subsp. *infantis* DSM 20088 [3], and to *B. kashiwanohense* DSM 21854 [39] and PV-20 [40]. Abyss assembly yielded 72 and 105 contigs (>500 bp) from 8.1 to 1.9 Mio reads for *B. longum* subsp. *suis* BSM11-5 and *B. longum* subsp. *infantis* TPY12-1, respectively (Table 5). For *B. longum* subsp. *suis* BSM11-5, the N50 was of 135.581 bp, and the largest contig had 298.614 bp. The N50 of *B. longum* subsp. *infantis* TPY12-1 was 94.696 bp with the largest contigs of 163.755 bp. ANI of *B. longum subsp. infantis* TPY12-1 compared to type strain was 98.4 %. *B. longum* subsp. *suis* type strain DSM 20211 and BSM11-5 had an ANI of 98.3 %, whereas ANI of *B. longum* subsp. *suis* BSM11-5 and *B. longum* subsp. *infantis* DSM 20088, or *B. longum* subsp. *longum* DSM 20019 was 96.6 and 96.7 %, respectively, confirming phylogenetic placement of this isolate based on partial 16S rRNA gene analysis (Additional file 1: Figure S1).

**Table 5:** Genome characteristics of *B. longum* subsp. *infantis, suis* and *B. kashiwanohense* strains used in this study

Strain	ID	Contigs	(Predicted) Genome size (Mbp)	GC-content	Coding sequences (RNAs)	Reference
*B. longum* subsp. *infantis*	DSM 20088	1	2.83	59.9	2432(91)	[3]
	TPY12-1	105	2.65	59.9	2371(58)	This study
*B. longum* subsp. *suis*	BSM11-5	72	2.61	59.9	2206(61)	This study
*B. kashiwanohense*	DSM 21854	1	2.34	56.3	1945	[39]
	PV20-2	1	2.37	56.1	1875(63)	[40]

Lo Cascio et al. [11] defined six gene regions related to gut adaption and HMO utilization which distinguished subspecies *B. longum* subsp. *longum* and *infantis* (urease, H1-H5). All six regions were present in the genome of *B. longum* subsp. *infantis* TPY12-1. *B. longum* subsp. *suis* BSM11-5 possessed an urea operon similar to *B. longum* subsp. *infantis*. This strain also harboured the LNB region (H5) as reported before for two *B. longum* subsp. *suis* isolates, and possessed parts of HMO utilization operon H1. However, *B. longum* subsp. *suis* BSM11-5 lacked additional α-fucosidase and sialidase containing gene regions H2-H4. Surprisingly, *B. kashiwanohense* DSM 21854 and PV20-2 also partly harboured H1 (Fig. 3), and *B. kashiwanohense* PV20-2 possessed an urea uptake and degradation operon with >90 % homology to *B. longum* subsp. *infantis* DSM 20088.

### Presence of α-fucosidase encoding genes in *B. longum* subsp. *infantis* TPY 12–1, *B. longum* subsp. *suis* BSM11-5*,* and *B. kashiwanohense*

Alpha-fucosidases, which catalyze the release of α-1-2, α-1-3, and α-1-4 linked fucose, are assigned to GH families 29 and 95 (GH29 and GH95). *B. longum* subsp. *infantis* DSM 20088 harbours four α-fucosidases: BLON_0248 (GH29), BLON_0426 (GH29), BLON_2335 (GH95) and BLON_2336 (GH29). *B. longum* subsp. *infantis* DSM 20088 also possesses BLON_0346, which is assigned to GH42 but can degrade Fucα1-2Gal [41]. Paralogs BLON_0248 and BLON_0426 are 95 % identical [41].

BlastP was used to identify homologues of the α-fucosidases of *B. longum* subsp. *infantis* DSM 20088 in the genomes of *B. longum* subsp. *infantis* TPY12-1, *B. longum* subsp. *suis* BSM11-5 and the two *B. kashiwanohense* strains (Table 6).

**Table 6:** Presence of α-fucosidases

	Strain				
	*B. longum* subsp. *infantis* DSM 20088	*B. longum* subsp. *infantis* TPY11-1	*B. longum* subsp. *suis* BSM 11-5	*B. kashiwanohense* DSM 21854	*B. kashiwanohense* PV20-2
Alpha-fucosidase	BLON_0248^a^	155 (99 %, 446/449)	–	BBKW_1714 (100 %, 449/449)	
	BLON_0426	–	–	–	
	BLON_0346	2339 (97 %, 247/254)	–	–	–
	BLON_2335^a^	2028 (98 %, 769/782)	229 (78 %, 607/783)	BBKW_1831 (77 %, 606/783)	AH68_10220 (78 %, 607/783)
	BLON_2336^a^	2029 (99 %, 475/478)	228 (88 %, 423/478)	BBKW_1832 (87 %, 417/478)	–

Alpha-fucosidases of *B. longum* subsp. *infantis* DSM 20088 and homologues present in the genomes of *B. longum* subsp. *infantis* TPY12-1, *B. longum* subsp. *suis* BSM11-5, and *B. kashiwanohense* strains

^a^A recent transcriptomic study investigated gene expression of *B. longum* subsp. *infantis* DSM 20088 in the presence of 2′-FL and 3′-FL, α-fucosidases that were overexpressed are indicated [34]

(−) not present, in brackets; similarity with *B. longum* subsp. *infantis* DSM 20088 α-fucosidases in AA

*B. longum* subsp. *infantis* TPY12-1 possessed homologues of BLON_0248/BLON_0426, BLON_0346, BLON_2335, and BLON_2336. *B. longum* subsp. *suis* BSM11-5 harboured two α-fucosidases highly similar to BLON_2335, and BLON_2336. *B. kashiwanohense* DSM 21854 and PV20-2 possessed three and one α-fucosidases, respectively, with high homology (>78 %) to *B. longum* subsp. *infantis* DSM 20088*.* BBKW_1714 of *B. kashiwanohense* DSM 21584 was flanked by a mobile element and was 100 % identical to BLON_0248/BLON_0426. *B. longum* subsp. *suis* BSM11-5, and *B. kashiwanohense* α-fucosidases were highly similar to each other (98–99 %) (Table 6). A search for conserved protein domains of every CAZyme family [27], identified no further GH29 or GH95 the genomes of *B. longum* subsp. *infantis* TPY12-1, *B. longum* subsp. *suis* BSM11-5, and *B. kashiwanohense* DSM 21854 and PV20-2.

Heterologously expressed BLON_0248, BLON_426, and BLON_0346 previously showed no activity on 2′-FL and 3′-FL [41], and BLON_2336 degraded only 3′-FL. BLON_2335 was able to degrade 2′-FL and 3′-FL [41] and homologues of BLON_2335 were present *B. longum* and *B. kashiwanohense* strains analysed here.

*B. kashiwanohense* has been rarely isolated infant stool and not yet from adults [20, 21], while *B. longum* subsp. *infantis* is considered an infant bifidobacteria species due to its adaption to degrade HMOs. The presence of highly similar genetic elements (region H1, α-fucosidases, urease operon) flanked by mobile elements indicate possible gene transfer between *B. longum* and *B. kashiwanohense* which suggests a co-evolutionary history between the two species that adapted *B. kashiwanohense* to the infant gut environment.

### Putative L-fucose degradation pathways in *B. longum* subsp. *infantis*, and *B. longum* subsp. *suis* BSM11-5

**Fig. 2:**
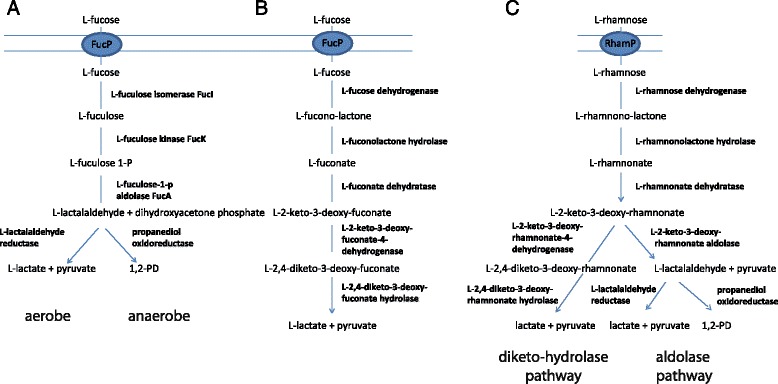
Comparison of L-fucose (**a**, **b**) and L-rhamnose (**c**) dedegradation pathways in *E. coli* (**a**), *X. campestris* (**b**), and *Sphingomonas* sp. (**c**) extracted from Boronat and Aguilar [30], Yew et al. [44], and Watanabe and Makino [45]

Strains of *B. longum* subsp. *infantis*, and *B. longum* subsp. *suis* BSM 11–5 used the L-fucose moiety of 2′-FL and 3′-FL to form 1,2-PD. However, the enzymatic pathway of *Bifidobacterium* sp. L-fucose metabolism is not known. Microorganisms can metabolize desoxyhexoses such as fucose by two pathways involving phosphorylated and non-phosphorylated intermediates (Fig. 2). For example in *E. coli, Roseburia inulivorans,* and *Lactobacillus rhamnosus* fucose isomerase FucI, fucose kinase FucK and fucose aldolase FucA are responsible for L-fucose to L-lactaldehyde transformation under anaerobic conditions [29, 30, 42]. L-rhamnose is metabolized via a similar enzymatic pathway [30]. For *Campylobacter jejuni* and *Xanthomonas campestris*, another pathway has been described with non-phosphorylated intermediates [43, 44]. Here L-fucose is internalized via the activity of a permease and metabolized to L-lactate and pyruvate by the activity of L-fucose mutarotase, L-fucose dehydrogenase, L-fuconolactone hydrolase, L-fuconate dehydratase, L-2-keto-3-deoxy-fuconate hydrolase, and a L-2,4-diketo-3-deoxy-fuconate hydrolase (Fig. 2, [43, 44]).

*B. longum* subsp. *infantis* DSM 20088 lacks the genes encoding proteins to use fucose via phosphorylation [3]. FucI, FucK and FucA were also not detected in the other genomes analysed here. To investigate whether bifidobacteria might utilize L-fucose similar to *X. campestris,* we searched for the corresponding proteins of *X. campestris* in bifidobacteria genomes using BlastP, and also collected enzymes related to fucose metabolism that were annotated by RAST.

*B. longum* subsp. *infantis* DSM 20088 and TPY11-1, and *B. longum* subsp. *suis* BSM11-5 possessed putative L-fucose dehydrogenases, a L-fuconolactone hydrolase, L-fuconate dehydratases, L-2-keto-3-deoxy-fuconate hydrolases, and a L-2,4-diketo-3-deoxy-fuconate hydrolase with homologies ranging from 24 to 56 % AA similarity to the enzymes of the *X. campestris* (Table 7). A homologue of the L-fucose mutarotase of *X. campestris* (XCC4070) was not detected; however, putative L-fucose mutarotases encompassing the conserved RbsD/FucU transport protein family domain were identified by RAST (Table 7).

**Table 7:**
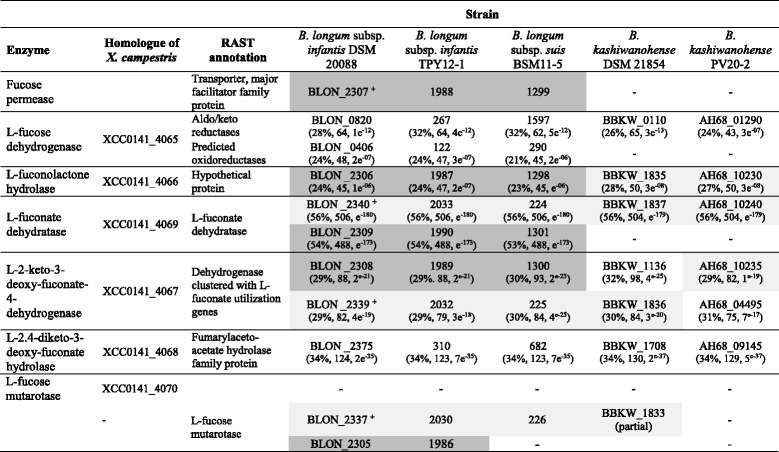
Identification of *B. longum* and *B. kashiwanohense* genes related to L-fucose metabolism

L-fucose related genes were identified by blastP search of homologous proteins of *X. campestris,* and by annotation by RAST using default settings. Shown are gene ID and in brackets bit scores and *e*-values of the obtained hits. Genes encoding these enzymes were predominantly located on two genomic regions shaded in light grey (region 1) and dark grey (region 2)

^**+**^A recent transcriptomic study investigated gene expression of *B. longum* subsp. *infantis* DSM 20088 in the presence of 2′-FL and 3′-FL, α-fucosidases that were overexpressed are indicated [34]

**Fig. 3:**
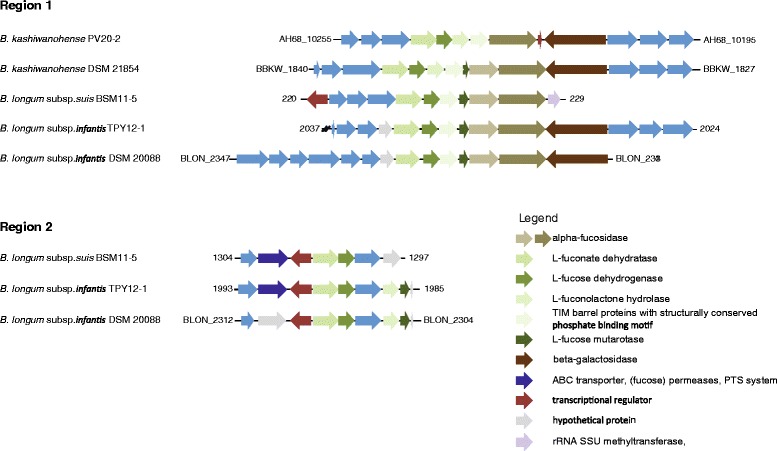
Genomic regions encompassing genes putatively involved in fucose degradation in *B. longum* and *B. kashiwanohense* strains. The gene cluster of region 1 also contained genes encoding the α-fucosidases BLON_2335 and BLON_2336 and is part of the *B. longum* subsp. *infantis* HMO utilization operon H1 [3, 11] (not drawn according to scale)

The majority of genes was located on two genomic regions (Fig. 3). In contrast, in *X. campestris* all responsible genes were located on an operon XCC4065-XCC4070 [44]. Region 1 encompassed a L-fucose mutarotase, a L-2-keto-3-deoxy-fuconate hydrolase, and a L-fuconate dehydratase (Fig. 3). The gene cluster of region 1 also contained genes encoding the α-fucosidases BLON_2335 and BLON_2336 and is part of the *B. longum* subsp. *infantis* HMO utilization operon H1 [3, 11]. A possible L-fuconolactone hydrolase, and paralogs of fuconate dehydratase and L-fucose mutarotase were located elsewhere on the genome in close proximity to a putative fucose permease (Fig. 3).

Expression of the fuconate dehydratase of region 1, and of the putative L-2-keto-3-deoxy-fuconate hydrolase was recently reported to be upregulated when *B. longum* subsp. *infantis* DSM 20088 was grown in the presence of fucosyllactose providing strong support to the proposition that L-fucose is metabolized via this pathway [34].

*B. kashiwanohense* DSM 21854 and PV20-2 only possessed a gene segment similar to region 1 with a truncated L-fucose-mutarotase, and lacked region 2 which encompassed the fucose permease. This might be the reason why strains of *B. kashiwanohense* were not able to utilize L-fucose.

For *X. campestris*, lactate and pyruvate, but not 1,2-PD, were determined as final metabolites of fucose fermentation. Watanabe and Makino [45] however described a modified non-phosphorylated L-rhamnose pathway which would yield L-lactaldehyde from L-2-keto-3-deoxyrhamnonate. The transformation of L-2-keto-3-deoxyrhamnonate to L-lactaldehyde and pyruvate was catalyzed by a L-2-keto-3-deoxyrhamnonate aldolase. A possible candidate gene could be the RAST annotated 4-hydroxy-tetrahydrodipicolinate synthase possessing a structurally conserved phosphate binding motif. 4-hydroxy-tetrahydrodipicolinate synthase encoding genes were located in region 1 of all strains investigated, *B. longum* subsp. *infantis* strains possessed a paralog in region 2*.* 4-hydroxy-tetrahydrodipicolinate synthases reversibly catalyze the formation of 4-hydroxy-2,3,4,5-tetrahydrodipicolinate from pyruvate and L-aspartate-4-semialdehyde [46].

Taken together these results suggest that *B. longum* subsp. *infantis* TPY12-1, DSM 20088 and *B. longum* subsp. *suis* BSM11-5 metabolize fucose via a pathway with non-phosphorylated intermediates as previously described for *Campylobacter* sp. and *X. campestris.*

## Conclusion

Here we observed that the ability to degrade fucosyllactose is a trait of various bifidobacteria species. We identified strains of *B. longum* subsp. *infantis* and an isolate of *B. longum* subsp. *suis* were able to degrade fucosyllactose and L-fucose to form 1,2-PD. We propose that bifidobacteria degrade L-fucose via a pathway with non-phosphorylated intermediates as described for desoxyhexoses before.

## Additional file

Additional file 1: Figure S1. Phylogenetic tree of partial (772 nt) 16S rRNA genes of strains used in this study Partial 16S rRNA gene sequences used to calculate phylogenetic tree. (DOC 2554 kb)
