# Supplementary material for: Fucosyllactose and L-fucose utilization of infant Bifidobacterium longum and Bifidobacterium kashiwanohense
Source: BMC Microbiol. 2016 Oct 26;16:248. doi: 10.1186/s12866-016-0867-4 (PMC5080750; doi:10.1186/s12866-016-0867-4)
Supplement: Additional file 1: Figure S1. — Phylogenetic tree of partial (772 nt) 16S rRNA genes of strains used in this study Partial 16S rRNA gene sequences used to calculate phylogenetic tree. (DOC 2554 kb) [file 12866_2016_867_MOESM1_ESM.doc]

Supplementary material for

**Fucosyllactose and L-fucose utilization of infant *Bifidobacterium longum* and *Bifidobacterium kashiwanohense***

Vera Bunesova^1,2^, Christophe Lacroix^1^, Clarissa Schwab^1,*^

^1^Laboratory of Food Biotechnology, Institute of Food, Nutrition and Health, ETH Zürich, Schmelzbergstrasse 7, 8092, Zürich, Switzerland

^2^Department of Microbiology, Nutrition and Dietetics, Faculty of Agrobiology, Food and Natural Resources, Czech University of Life Sciences Prague, Kamycka 129, Prague 6, 165 21, Czech Republic

**Corresponding author*:

Clarissa Schwab, Laboratory of Food Biotechnology, Institute of Food, Nutrition and Health, Department of Health Sciences and Technology, ETH Zürich, Schmelzbergstrasse 7, 8092 Zürich, Switzerland

E-mail: [clarissa.schwab@hest.ethz.ch](mailto:clarissa.schwab@hest.ethz.ch)

**Content:**

Supplementary Figure

16S rRNA gene sequences


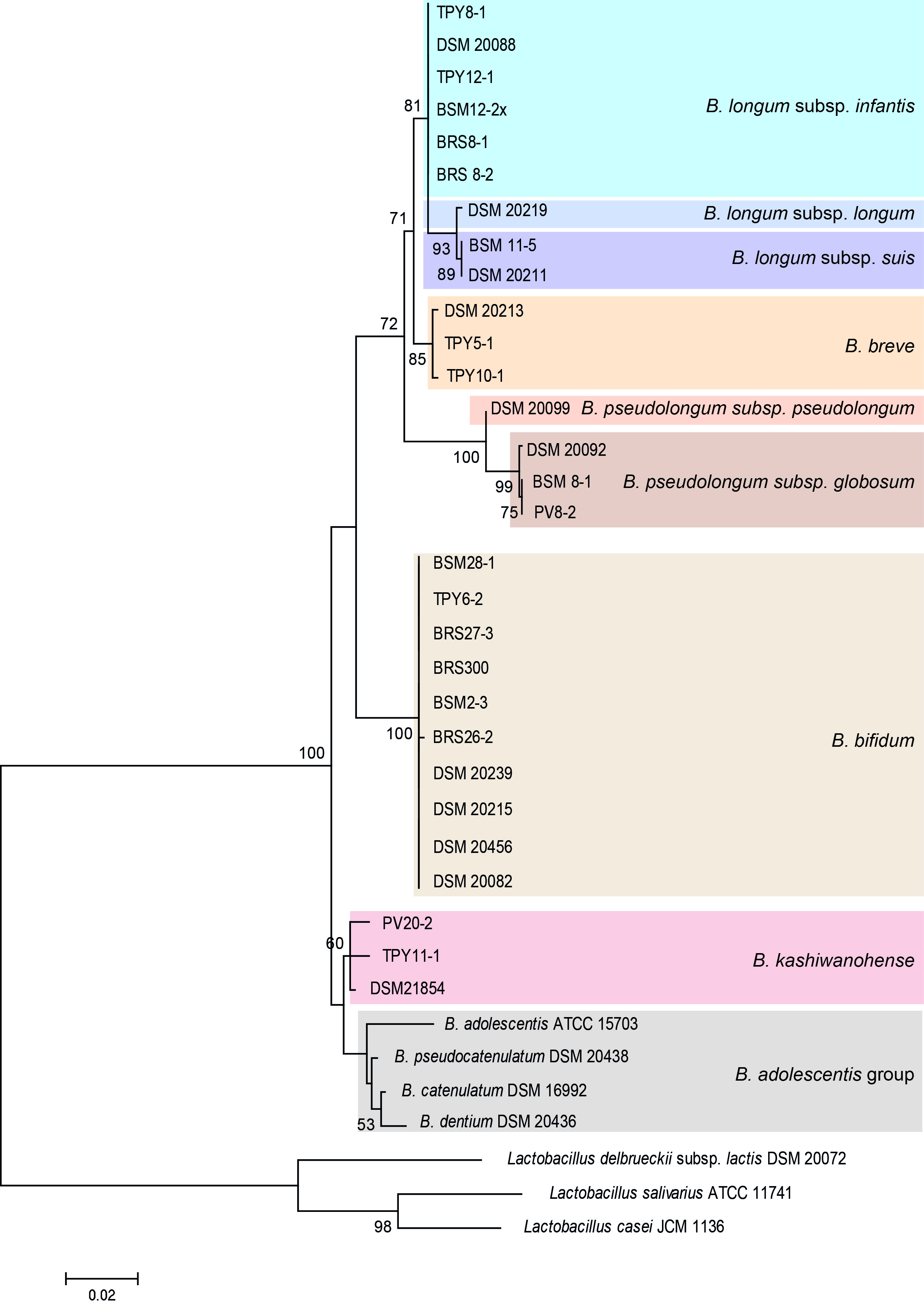


**Figure S1.** Phylogenetic tree of partial (772 nt) 16S rRNA genes of strains used in this study

Partial 16S rRNA gene sequences used to calculate phylogenetic tree

**>BBIF BRS26-2**

TGTGAAAGTCCATCGCTTAACGGTGGATCTGCGCCGGGTACGGGCGGGCTGGAGTGCGGTAGGGGAGACTGGAATTCCCGGTGTAACGGTGGAATGTGTAGATATCGGGAAGAACACCGATGGCGAAGGCAGGTCTCTGGGCCGTCACTGACGCTGAGGAGCGAAAGCGTGGGGAGCGAACAGGATTAGATACCCTGGTAGTCCACGCCGTAAACGGTGGACGCTGGATGTGGGGCACGTTCCACGTGTTCCGTGTCGGAGCTAACGCGTTAAGCGTCCCGCCTGGGGAGTACGGCCGCAAGGCTAAAACTCAAAGAAATTGACGGGGGCCCGCACAAGCGGCGGAGCATGCGGATTAATTCGATGCAACGCGAAGAACCTTACCTGGGCTTGACATGTTCCCGACGACGCCAGAGATGGCGTTTCCCTTCGGGGCGGGTTCACAGGTGGTGCATGGTCGTCGTCAGCTCGTGTCGTGAGATGTTGGGTTAAGTCCCGCAACGAGCGCAACCCTCGCCCCGTGTTGCCAGCACGTTATGGTGGGAACTCACGGGGGACCGCCGGGGTTAACTCGGAGGAAGGTGGGGATGACGTCAGATCATCATGCCCCTTACGTCCAGGGCTTCACGCATGCTACAATGGCCGGTACAGCGGGATGCGACATGGCGACATGGAGCGGATCCCTGAAAACCGGTCTCAGTTCGGATCGGAGCCTGCAACCCGGCTCCGGAAGGCGGAGTCGTTAAGTAATCGCGATCA

**>BBIF BSM2-3**

TGTGAAAGTCCATCGCTTAACGGTGGATCTGCGCCGGGTACGGGCGGGCTGGAGTGCGGTAGGGGAGACTGGAATTCCCGGTGTAACGGTGGAATGTGTAGATATCGGGAAGAACACCGATGGCGAAGGCAGGTCTCTGGGCCGTCACTGACGCTGAGGAGCGAAAGCGTGGGGAGCGAACAGGATTAGATACCCTGGTAGTCCACGCCGTAAACGGTGGACGCTGGATGTGGGGCACGTTCCACGTGTTCCGTGTCGGAGCTAACGCGTTAAGCGTCCCGCCTGGGGAGTACGGCCGCAAGGCTAAAACTCAAAGAAATTGACGGGGGCCCGCACAAGCGGCGGAGCATGCGGATTAATTCGATGCAACGCGAAGAACCTTACCTGGGCTTGACATGTTCCCGACGACGCCAGAGATGGCGTTTCCCTTCGGGGCGGGTTCACAGGTGGTGCATGGTCGTCGTCAGCTCGTGTCGTGAGATGTTGGGTTAAGTCCCGCAACGAGCGCAACCCTCGCCCCGTGTTGCCAGCACGTTATGGTGGGAACTCACGGGGGACCGCCGGGGTTAACTCGGAGGAAGGTGGGGATGACGTCAGATCATCATGCCCCTTACGTCCAGGGCTTCACGCATGCTACAATGGCCGGTACAGCGGGATGCGACATGGCGACATGGAGCGGACCCTGAAAACCGGTCTCAGTTCGGATCGGAGCCTGCAACCCGGCTCCGTGAAGGCGGAGTCGCTAGTAATCGCGATCA

**>BBIF** **BRS300**

TGTGAAAGTCCATCGCTTAACGGTGGATCTGCGCCGGGTACGGGCGGGCTGGAGTGCGGTAGGGGAGACTGGAATTCCCGGTGTAACGGTGGAATGTGTAGATATCGGGAAGAACACCGATGGCGAAGGCAGGTCTCTGGGCCGTCACTGACGCTGAGGAGCGAAAGCGTGGGGAGCGAACAGGATTAGATACCCTGGTAGTCCACGCCGTAAACGGTGGACGCTGGATGTGGGGCACGTTCCACGTGTTCCGTGTCGGAGCTAACGCGTTAAGCGTCCCGCCTGGGGAGTACGGCCGCAAGGCTAAAACTCAAAGAAATTGACGGGGGCCCGCACAAGCGGCGGAGCATGCGGATTAATTCGATGCAACGCGAAGAACCTTACCTGGGCTTGACATGTTCCCGACGACGCCAGAGATGGCGTTTCCCTTCGGGGCGGGTTCACAGGTGGTGCATGGTCGTCGTCAGCTCGTGTCGTGAGATGTTGGGTTAAGTCCCGCAACGAGCGCAACCCTCGCCCCGTGTTGCCAGCACGTTATGGTGGGAACTCACGGGGGACCGCCGGGGTTAACTCGGAGGAAGGTGGGGATGACGTCAGATCATCATGCCCCTTACGTCCAGGGCTTCACGCATGCTACAATGGCCGGTACAGCGGGATGCGACATGGCGACATGGAGCGGATCCCTGAAAACCGGTCTCAGTTCGGATCGGAGCCTGCAACCCGGCTCCGTGAAGGCGGAGTCGCTA-GTAATCGCGGATCA

**>BBIF BRS27-3**

TGTGAAAGTCCATCGCTTAACGGTGGATCTGCGCCGGGTACGGGCGGGCTGGAGTGCGGTAGGGGAGACTGGAATTCCCGGTGTAACGGTGGAATGTGTAGATATCGGGAAGAACACCGATGGCGAAGGCAGGTCTCTGGGCCGTCACTGACGCTGAGGAGCGAAAGCGTGGGGAGCGAACAGGATTAGATACCCTGGTAGTCCACGCCGTAAACGGTGGACGCTGGATGTGGGGCACGTTCCACGTGTTCCGTGTCGGAGCTAACGCGTTAAGCGTCCCGCCTGGGGAGTACGGCCGCAAGGCTAAAACTCAAAGAAATTGACGGGGGCCCGCACAAGCGGCGGAGCATGCGGATTAATTCGATGCAACGCGAAGAACCTTACCTGGGCTTGACATGTTCCCGACGACGCCAGAGATGGCGTTTCCCTTCGGGGCGGGTTCACAGGTGGTGCATGGTCGTCGTCAGCTCGTGTCGTGAGATGTTGGGTTAAGTCCCGCAACGAGCGCAACCCTCGCCCCGTGTTGCCAGCACGTTATGGTGGGAACTCACGGGGGACCGCCGGGGTTAACTCGGAGGAAGGTGGGGATGACGTCAGATCATCATGCCCCTTACGTCCAGGGCTTCACGCATGCTACAATGGCCGGTACAGCGGGATGCGACATGGCGACATGGAGCGGATCCCTGAAAACCGGTCTCAGTTCGGATCGGAGCCTGCAACCCGGCTCCGTGAAGGCGGAGTCGCTAGTAATCGCGGATCA

**>BBIF BSM28-1**

TGTGAAAGTCCATCGCTTAACGGTGGATCTGCGCCGGGTACGGGCGGGCTGGAGTGCGGTAGGGGAGACTGGAATTCCCGGTGTAACGGTGGAATGTGTAGATATCGGGAAGAACACCGATGGCGAAGGCAGGTCTCTGGGCCGTCACTGACGCTGAGGAGCGAAAGCGTGGGGAGCGAACAGGATTAGATACCCTGGTAGTCCACGCCGTAAACGGTGGACGCTGGATGTGGGGCACGTTCCACGTGTTCCGTGTCGGAGCTAACGCGTTAAGCGTCCCGCCTGGGGAGTACGGCCGCAAGGCTAAAACTCAAAGAAATTGACGGGGGCCCGCACAAGCGGCGGAGCATGCGGATTAATTCGATGCAACGCGAAGAACCTTACCTGGGCTTGACATGTTCCCGACGACGCCAGAGATGGCGTTTCCCTTCGGGGCGGGTTCACAGGTGGTGCATGGTCGTCGTCAGCTCGTGTCGTGAGATGTTGGGTTAAGTCCCGCAACGAGCGCAACCCTCGCCCCGTGTTGCCAGCACGTTATGGTGGGAACTCACGGGGGACCGCCGGGGTTAACTCGGAGGAAGGTGGGGATGACGTCAGATCATCATGCCCCTTACGTCCAGGGCTTCACGCATGCTACAATGGCCGGTACAGCGGGATGCGACATGGCGACATGGAGCGGATCCCTGAAAACCGGTCTCAGTTCGGATCGGAGCCTGCAACCCGGCTCCGTGAAGGCGGAGTCGCTA-GTAATCGCGGATCA

**>BBIF TPY6-2**

TGTGAAAGTCCATCGCTTAACGGTGGATCTGCGCCGGGTACGGGCGGGCTGGAGTGCGGTAGGGGAGACTGGAATTCCCGGTGTAACGGTGGAATGTGTAGATATCGGGAAGAACACCGATGGCGAAGGCAGGTCTCTGGGCCGTCACTGACGCTGAGGAGCGAAAGCGTGGGGAGCGAACAGGATTAGATACCCTGGTAGTCCACGCCGTAAACGGTGGACGCTGGATGTGGGGCACGTTCCACGTGTTCCGTGTCGGAGCTAACGCGTTAAGCGTCCCGCCTGGGGAGTACGGCCGCAAGGCTAAAACTCAAAGAAATTGACGGGGGCCCGCACAAGCGGCGGAGCATGCGGATTAATTCGATGCAACGCGAAGAACCTTACCTGGGCTTGACATGTTCCCGACGACGCCAGAGATGGCGTTTCCCTTCGGGGCGGGTTCACAGGTGGTGCATGGTCGTCGTCAGCTCGTGTCGTGAGATGTTGGGTTAAGTCCCGCAACGAGCGCAACCCTCGCCCCGTGTTGCCAGCACGTTATGGTGGGAACTCACGGGGGACCGCCGGGGTTAACTCGGAGGAAGGTGGGGATGACGTCAGATCATCATGCCCCTTACGTCCAGGGCTTCACGCATGCTACAATGGCCGGTACAGCGGGATGCGACATGGCGACATGGAGCGGATCCCTGAAAACCGGTCTCAGTTCGGATCGGAGCCTGCAACCCGGCTCCGTGAAGGCGGAGTCGCTAGTAATCGCGGATCA

**>BBRE TPY10-1**

TGTGAAAGTCCATCGCTTAACGGTGGATCCGCGCCGGGTACGGGCGGGCTTGAGTGCGGTAGGGGAGACTGGAATTCCCGGTGTAACGGTGGAATGTGTAGATATCGGGAAGAACACCAATGGCGAAGGCAGGTCTCTGGGCCGTTACTGACGCTGAGGAGCGAAAGCGTGGGGAGCGAACAGGATTAGATACCCTGGTAGTCCACGCCGTAAACGGTGGATGCTGGATGTGGGGCCCGTTCCACGGGTTCCGTGTCGGAGCTAACGCGTTAAGCATCCCGCCTGGGGAGTACGGCCGCAAGGCTAAAACTCAAAGAAATTGACGGGGGCCCGCACAAGCGGCGGAGCATGCGGATTAATTCGATGCAACGCGAAGAACCTTACCTGGGCTTGACATGTTCCCGACGACCCCAGAGATGGGGTTTCCCTTCGGGGCGGGTTCACAGGTGGTGCATGGTCGTCGTCAGCTCGTGTCGTGAGATGTTGGGTTAAGTCCCGCAACGAGCGCAACCCTCGCCCCGTGTTGCCAGCGGATTATGCCGGGAACTCACGGGGGACCGCCGGGGTTAACTCGGAGGAAGGTGGGGATGACGTCAGATCATCATGCCCCTTACGTCCAGGGCTTCACGCATGCTACAATGGCCGGTACAACGGGATGCGACAGTGCGAGCTGGAGCGGATCCCTGAAAACCGGTCTCAGTTCGGATCGCAGTCTGCAACTCGACTGCGTGAAGGCGGAGTCGCTA-GTAATCGCGAATCA

**>BBRE BSM1-2**

TGTGAAAGTCCATCGCTTAACGGTGGATCCGCGCCGGGTACGGGCGGGCTTGAGTGCGGTAGGGGAGACTGGAATTCCCGGTGTAACGGTGGAATGTGTAGATATCGGGAAGAACACCAATGGCGAAGGCAGGTCTCTGGGCCGTTACTGACGCTGAGGAGCGAAAGCGTGGGGAGCGAACAGGATTAGATACCCTGGTAGTCCACGCCGTAAACGGTGGATGCTGGATGTGGGGCCCGTTCCACGGGTTCCGTGTCGGAGCTAACGCGTTAAGCATCCCGCCTGGGGAGTACGGCCGCAAGGCTAAAACTCAAAGAAATTGACGGGGGCCCGCACAAGCGGCGGAGCATGCGGATTAATTCGATGCAACGCGAAGAACCTTACCTGGGCTTGACATGTTCCCGACGACCCCAGAGATGGGGTTTCCCTTCGGGGCGGGTTCACAGGTGGTGCATGGTCGTCGTCAGCTCGTGTCGTGAGATGTTGGGTTAAGTCCCGCAACGAGCGCAACCCTCGCCCCGTGTTGCCAGCGGATTATGCCGGGAACTCACGGGGGACCGCCGGGGTTAACTCGGAGGAAGGTGGGGATGACGTCAGATCATCATGCCCCTTACGTCCAGGGCTTCACGCATGCTACAATGGCCGGTACAACGGGATGCGACAGTGCGAGCTGGAGCGGATCCCTGAAAACCGGTCTCAGTTCGGATCGCAGTCTGCAACTCGACTGCGTGAAGGCGGAGTCGCTA-GTAATCGCGAATCA

**>BBRE TPY5-1**

TGTGAAAGTCCATCGCTTAACGGTGGATCCGCGCCGGGTACGGGCGGGCTTGAGTGCGGTAGGGGAGACTGGAATTCCCGGTGTAACGGTGGAATGTGTAGATATCGGGAAGAACACCAATGGCGAAGGCAGGTCTCTGGGCCGTTACTGACGCTGAGGAGCGAAAGCGTGGGGAGCGAACAGGATTAGATACCCTGGTAGTCCACGCCGTAAACGGTGGATGCTGGATGTGGGGCCCGTTCCACGGGTTCCGTGTCGGAGCTAACGCGTTAAGCATCCCGCCTGGGGAGTACGGCCGCAAGGCTAAAACTCAAAGAAATTGACGGGGGCCCGCACAAGCGGCGGAGCATGCGGATTAATTCGATGCAACGCGAAGAACCTTACCTGGGCTTGACATGTTCCCGACGATCCCAGAGATGGGGTTTCCCTTCGGGGCGGGTTCACAGGTGGTGCATGGTCGTCGTCAGCTCGTGTCGTGAGATGTTGGGTTAAGTCCCGCAACGAGCGCAACCCTCGCCCCGTGTTGCCAGCGGATTATGCCGGGAACTCACGGGGGACCGCCGGGGTTAACTCGGAGGAAGGTGGGGATGACGTCAGATCATCATGCCCCTTACGTCCAGGGCTTCACGCATGCTACAATGGCCGGTACAACGGGATGCGACAGTGCGAGCTGGAGCGGATCCCTGAAAACCGGTCTCAGTTCGGATCGCAGTCTGCAACTCGACTGCGTGAAGGCGGAGTCGCTA-GTAATCGCGAATCA

**>BBRE (N12-NF3-i3)**

TGTGAAAGTCCATCGCTTAACGGTGGATCCGCGCCGGGTACGGGCGGGCTTGAGTGCGGTAGGGGAGACTGGAATTCCCGGTGTAACGGTGGAATGTGTAGATATCGGGAAGAACACCAATGGCGAAGGCAGGTCTCTGGGCCGTTACTGACGCTGAGGAGCGAAAGCGTGGGGAGCGAACAGGATTAGATACCCTGGTAGTCCATGCCGTAAACGGTGGATGCTGGATGTGGGGCCCGTTCCACGGGTTCCGTGTCGGAGCTAACGCGTTAAGCATCCCGCCTGGGGAGTACGGCCGCAAGGCTAAAACTCAAAGAAATTGACGGGGGCCCGCACAAGCGGCGGAGCATGCGGATTAATTCGATGCAACGCGAAGAACCTTACCTGGGCTTGACATGTTCCCGACGATCCCAGAGATGGGGTTTCCCTTCGGGGCGGGTTCACAGGTGGTGCATGGTCGTCGTCAGCTCGTGTCGTGAGATGTTGGGTTAAGTCCCGCAACGAGCGCAACCCTCGCCCCGTGTTGCCAGCGGATTGTGCCGGGAACTCACGGGGGACCGCCGGGGTTAACTCGGAGGAAGGTGGGGATGACGTCAGATCATCATGCCCCTTACGTCCAGGGCTTCACGCATGCTACAATGGCCGGTACAACGGGATGCGACAGTGCGAGCTGGAGCGGATCCCTGAAAACCGGTCTCAGTTCGGATCGCAGTCTGCAACTCGACTGCGTGAAGGCGGAGTCGCTAGTAATCGCGAATCA

**>BKASH PV20-2**

TGTGAAAGTCCATCGCTTAACGGTGGATCCGCGCCGGGTACGGGCGGGCTTGAGTGCGGTAGGGGAGACTGGAATTCCCGGTGTAACGGTGGAATGTGTAGATATCGGGAAGAACACCAATGGCGAAGGCAGGTCTCTGGGCCGTTACTGACGCTGAGGAGCGAAAGCGTGGGGAGCGAACAGGATTAGATACCCTGGTAGTCCACGCCGTAAACGGTGGATGCTGGATGTGGGGCCCGTTCCACGGGTTCCGTGTCGGAGCTAACGCGTTAAGCATCCCGCCTGGGGAGTACGGCCGCAAGGCTAAAACTCAAAGAAATTGACGGGGGCCCGCACAAGCGGCGGAGCATGCGGATTAATTCGATGCAACGCGAAGAACCTTACCTGGGCTTGACATGTTCCCGACCGCTCCAGAGATGGGGCTTCCCTTCGGGGCGGGTTCACAGGTGGTGCATGGTCGTCGTCAGCTCGTGTCGTGAGATGTTGGGTTAAGTCCCGCAACGAGCGCAACCCTCGCCCTGTGTTGCCAGCACATGATGGTGGGAACTCACGGGGGACCGCCGGGGTCAACTCGGAGGAAGGTGGGGATGACGTCAGATCATCATGCCCCTTACGTCCAGGGCTTCACGCATGCTACAATGGCCGGTACAACGGGATGCGACACGGCGACGTGGAGCGGATCCCTGAAAACCGGTCTCAGTTCGGATTGGAGTCTGCAACCCGACTCCATGAAGGCGGAGTCGCTA-GTAATCGCGGATCA

**>BKASH TPY11-1**

TGTGAAAGTCCATCGCTTAACGGTGGATCTGCGCCGGGTACGGGCGGGCTGGAGTGCGGTAGGGGAGACTGGAATTCCCGGTGTAACGGTGGAATGTGTAGATATCGGGAAGAACACCAATGGCGAAGGCAGGTCTCTGGGCCGTTACTGACGCTGAGGAGCGAAAGCGTGGGGAGCGAACAGGATTAGATACCCTGGTAGTCCACGCCGTAAACGGTGGATGCTGGATGTGGGGCCCGTTCCACGGGTTCCGTGTCGGAGCTAACGCGTTAAGCATCCCGCCTGGGGAGTACGGCCGCAAGGCTAAAACTCAAAGAAATTGACGGGGGCCCGCACAAGCGGCGGAGCATGCGGATTAATTCGATGCAACGCGAAGAACCTTACCTGGGCTTGACATGTTCCCGACCGCTCCAGAGATGGGGCTTCCCTTCGGGGCGGGTTCACAGGTGGTGCATGGTCGTCGTCAGCTCGTGTCGTGAGATGTTGGGTTAAGTCCCGCAACGAGCGCAACCCTCGCCCTGTGTTGCCAGCGCGTCATGGCGGGAACTCACGGGGGACCGCCGGGGTCAACTCGGAGGAAGGTGGGGATGACGTCAGATCATCATGCCCCTTACGTCCAGGGCTTCACGCATGCTACAATGGCCGGTACAACGGGATGCGACATGGCGACATGGAGCGGATCCCTGAAAACCGGTCTCAGTTCGGATTGGAGTCTGCAACCCGACTCCATGAAGGCGGAGTCGCTAGTAATCGCGGATCA

**>BLON BRS8-2**

TGTGAAAGTCCATCGCTTAACGGTGGATCCGCGCCGGGTACGGGCGGGCTTGAGTGCGGTAGGGGAGACTGGAATTCCCGGTGTAACGGTGGAATGTGTAGATATCGGGAAGAACACCAATGGCGAAGGCAGGTCTCTGGGCCGTTACTGACGCTGAGGAGCGAAAGCGTGGGGAGCGAACAGGATTAGATACCCTGGTAGTCCACGCCGTAAACGGTGGATGCTGGATGTGGGGCCCGTTCCACGGGTTCCGTGTCGGAGCTAACGCGTTAAGCATCCCGCCTGGGGAGTACGGCCGCAAGGCTAAAACTCAAAGAAATTGACGGGGGCCCGCACAAGCGGCGGAGCATGCGGATTAATTCGATGCAACGCGAAGAACCTTACCTGGGCTTGACATGTTCCCGACGATCCCAGAGATGGGGTTTCCCTTCGGGGCGGGTTCACAGGTGGTGCATGGTCGTCGTCAGCTCGTGTCGTGAGATGTTGGGTTAAGTCCCGCAACGAGCGCAACCCTCGCCCCGTGTTGCCAGCGGATTGTGCCGGGAACTCACGGGGGACCGCCGGGGTTAACTCGGAGGAAGGTGGGGATGACGTCAGATCATCATGCCCCTTACGTCCAGGGCTTCACGCATGCTACAATGGCCGGTACAACGGGATGCGACGCGGCGACGCGGAGCGGATCCCTGAAAACCGGTCTCAGTTCGGATCGCAGTCTGCAACTCGACTGCGTGAAGGCGGAGTCGCTA-GTAATCGCGAATCA

**>BLON TPY12-1**

TGTGAAAGTCCATCGCTTAACGGTGGATCCGCGCCGGGTACGGGCGGGCTTGAGTGCGGTAGGGGAGACTGGAATTCCCGGTGTAACGGTGGAATGTGTAGATATCGGGAAGAACACCAATGGCGAAGGCAGGTCTCTGGGCCGTTACTGACGCTGAGGAGCGAAAGCGTGGGGAGCGAACAGGATTAGATACCCTGGTAGTCCACGCCGTAAACGGTGGATGCTGGATGTGGGGCCCGTTCCACGGGTTCCGTGTCGGAGCTAACGCGTTAAGCATCCCGCCTGGGGAGTACGGCCGCAAGGCTAAAACTCAAAGAAATTGACGGGGGCCCGCACAAGCGGCGGAGCATGCGGATTAATTCGATGCAACGCGAAGAACCTTACCTGGGCTTGACATGTTCCCGACGATCCCAGAGATGGGGTTTCCCTTCGGGGCGGGTTCACAGGTGGTGCATGGTCGTCGTCAGCTCGTGTCGTGAGATGTTGGGTTAAGTCCCGCAACGAGCGCAACCCTCGCCCCGTGTTGCCAGCGGATTGTGCCGGGAACTCACGGGGGACCGCCGGGGTTAACTCGGAGGAAGGTGGGGATGACGTCAGATCATCATGCCCCTTACGTCCAGGGCTTCACGCATGCTACAATGGCCGGTACAACGGGATGCGACGCGGCGACGCGGAGCGGATCCCTGAAAACCGGTCTCAGTTCGGATCGCAGTCTGCAACTCGACTGCGGAAGGCGGAGTCGCTAGTAATCGCGAATCA

**>BLON BSM12-2x**

TGTGAAAGTCCATCGCTTAACGGTGGATCCGCGCCGGGTACGGGCGGGCTTGAGTGCGGTAGGGGAGACTGGAATTCCCGGTGTAACGGTGGAATGTGTAGATATCGGGAAGAACACCAATGGCGAAGGCAGGTCTCTGGGCCGTTACTGACGCTGAGGAGCGAAAGCGTGGGGAGCGAACAGGATTAGATACCCTGGTAGTCCACGCCGTAAACGGTGGATGCTGGATGTGGGGCCCGTTCCACGGGTTCCGTGTCGGAGCTAACGCGTTAAGCATCCCGCCTGGGGAGTACGGCCGCAAGGCTAAAACTCAAAGAAATTGACGGGGGCCCGCACAAGCGGCGGAGCATGCGGATTAATTCGATGCAACGCGAAGAACCTTACCTGGGCTTGACATGTTCCCGACGATCCCAGAGATGGGGTTTCCCTTCGGGGCGGGTTCACAGGTGGTGCATGGTCGTCGTCAGCTCGTGTCGTGAGATGTTGGGTTAAGTCCCGCAACGAGCGCAACCCTCGCCCCGTGTTGCCAGCGGATTGTGCCGGGAACTCACGGGGGACCGCCGGGGTTAACTCGGAGGAAGGTGGGGATGACGTCAGATCATCATGCCCCTTACGTCCAGGGCTTCACGCATGCTACAATGGCCGGTACAACGGGATGCGACGCGGCGACGCGGAGCGGATCCCTGAAAACCGGTCTCAGTTCGGATCGCAGTCTGCAACTCGACTGCGTGAAGGCGGAGTCGCTA-GTAATCGCGAATCA

**>BLON BRS8-1**

TGTGAAAGTCCATCGCTTAACGGTGGATCCGCGCCGGGTACGGGCGGGCTTGAGTGCGGTAGGGGAGACTGGAATTCCCGGTGTAACGGTGGAATGTGTAGATATCGGGAAGAACACCAATGGCGAAGGCAGGTCTCTGGGCCGTTACTGACGCTGAGGAGCGAAAGCGTGGGGAGCGAACAGGATTAGATACCCTGGTAGTCCACGCCGTAAACGGTGGATGCTGGATGTGGGGCCCGTTCCACGGGTTCCGTGTCGGAGCTAACGCGTTAAGCATCCCGCCTGGGGAGTACGGCCGCAAGGCTAAAACTCAAAGAAATTGACGGGGGCCCGCACAAGCGGCGGAGCATGCGGATTAATTCGATGCAACGCGAAGAACCTTACCTGGGCTTGACATGTTCCCGACGATCCCAGAGATGGGGTTTCCCTTCGGGGCGGGTTCACAGGTGGTGCATGGTCGTCGTCAGCTCGTGTCGTGAGATGTTGGGTTAAGTCCCGCAACGAGCGCAACCCTCGCCCCGTGTTGCCAGCGGATTGTGCCGGGAACTCACGGGGGACCGCCGGGGTTAACTCGGAGGAAGGTGGGGATGACGTCAGATCATCATGCCCCTTACGTCCAGGGCTTCACGCATGCTACAATGGCCGGTACAACGGGATGCGACGCGGCGACGCGGAGCGGATCCCTGAAAACCGGTCTCAGTTCGGATCGCAGTCTGCAACTCGACTGCGTGAAGGCGGAGTCGCTA-GTAATCGCGAATCA

**>BLON BSM11-5**

TGTGAAAGTCCATCGCTTAACGGTGGATCCGCGCCGGGTACGGGCGGGCTTGAGTGCGGTAGGGGAGACTGGAATTCCCGGTGTAACGGTGGAATGTGTAGATATCGGGAAGAACACCAATGGCGAAGGCAGGTCTCTGGGCCGTTACTGACGCTGAGGAGCGAAAGCGTGGGGAGCGAACAGGATTAGATACCCTGGTAGTCCACGCCGTAAACGGTGGATGCTGGATGTGGGGCCCGTTCCACGGGTTCCGTGTCGGAGCTAACGCGTTAAGCATCCCGCCTGGGGAGTACGGCCGCAAGGCTAAAACTCAAAGAAATTGACGGGGGCCCGCACAAGCGGCGGAGCATGCGGATTAATTCGATGCAACGCGAAGAACCTTACCTGGGCTTGACATGTTCCCGACGGCCGTAGAGATACGGCTTCCCTTCGGGGCGGGTTCACAGGTGGTGCATGGTCGTCGTCAGCTCGTGTCGTGAGATGTTGGGTTAAGTCCCGCAACGAGCGCAACCCTCGCCCCGTGTTGCCAGCGGATTGTGCCGGGAACTCACGGGGGACCGCCGGGGTTAACTCGGAGGAAGGTGGGGATGACGTCAGATCATCATGCCCCTTACGTCCAGGGCTTCACGCATGCTACAATGGCCGGTACAACGGGATGCGACGCGGCGACGCGGAGCGGATCCCTGAAAACCGGTCTCAGTTCGGATCGCAGTCTGCAACTCGACTGCG-GAAGGCGGAGTCGCTA-GTAATCGCGAATCA

**>BLON TPY8-1**

TGTGAAAGTCCATCGCTTAACGGTGGATCCGCGCCGGGTACGGGCGGGCTTGAGTGCGGTAGGGGAGACTGGAATTCCCGGTGTAACGGTGGAATGTGTAGATATCGGGAAGAACACCAATGGCGAAGGCAGGTCTCTGGGCCGTTACTGACGCTGAGGAGCGAAAGCGTGGGGAGCGAACAGGATTAGATACCCTGGTAGTCCACGCCGTAAACGGTGGATGCTGGATGTGGGGCCCGTTCCACGGGTTCCGTGTCGGAGCTAACGCGTTAAGCATCCCGCCTGGGGAGTACGGCCGCAAGGCTAAAACTCAAAGAAATTGACGGGGGCCCGCACAAGCGGCGGAGCATGCGGATTAATTCGATGCAACGCGAAGAACCTTACCTGGGCTTGACATGTTCCCGACGATCCCAGAGATGGGGTTTCCCTTCGGGGCGGGTTCACAGGTGGTGCATGGTCGTCGTCAGCTCGTGTCGTGAGATGTTGGGTTAAGTCCCGCAACGAGCGCAACCCTCGCCCCGTGTTGCCAGCGGATTGTGCCGGGAACTCACGGGGGACCGCCGGGGTTAACTCGGAGGAAGGTGGGGATGACGTCAGATCATCATGCCCCTTACGTCCAGGGCTTCACGCATGCTACAATGGCCGGTACAACGGGATGCGACGCGGCGACGCGGAGCGGATCCCTGAAAACCGGTCTCAGTTCGGATCGCAGTCTGCAACTCGACTGCGTGAAGGCGGAGTCGCTA-GTAATCGCGAATCA

**>BPSE PV8-2** GTGAAAGTCCATCGCTTAACGGTGGATCCGCGCCGGGTACGGGCGGGCTTGAGTGCGGTAGGGGAGACTGGAATTCCCGGTGTAACGGTGGAATGTGTAGATATCGGGAAGAACACCAATGGCGAAGGCAGGTCTCTGGGCCGTTACTGACGCTGAGGAGCGAAAGCGTGGGGAGCGAACAGGATTAGATACCCTGGTAGTCCACGCCGTAAACGGTGGATGCTGGATGTGGGGCCCTTTTTCCGGGTCCTGTGTCGGAGCTAACGCGTTAAGCATCCCGCCTGGGGAGTACGGCCGCAAGGCTAAAACTCAAAGAAATTGACGGGGGCCCGCACAAGCGGCGGAGCATGCGGATTAATTCGATGCAACGCGAAGAACCTTACCTGGGCTTGACATGTGCCGGACGCCCGCGGAGACGCGGGTTCCCTTCGGGGCCGGTTCACAGGTGGTGCATGGTCGTCGTCAGCTCGTGTCGTGAGATGTTGGGTTAAGTCCCGCAACGAGCGCAACCCTCGCCGCGTGTTGCCAGCGGGTCATGCCGGGAACTCACGTGGGACCGCCGGGGTTAACTCGGAGGAAGGTGGGGATGACGTCAGATCATCATGCCCCTTACGTCCAGGGCTTCACGCATGCTACAATGGCCGGTACAACGGGGTGCGACACGGTGACGTGGGGCGGATCCCTGAAAACCGGTCTCAGTTCGGATCGCAGTCTGCAACTCGACTGCGTGAAGGTGGAGTCGCTA-GTAATCGCGGATCA

**>BPSE BSM8-3**

TGTGAAAGTCCATCGCTTAACGGTGGATCCGCGCCGGGTACGGGCGGGCTTGAGTGCGGTAGGGGAGACTGGAATTCCCGGTGTAACGGTGGAATGTGTAGATATCGGGAAGAACACCAATGGCGAAGGCAGGTCTCTGGGCCGTTACTGACGCTGAGGAGCGAAAGCGTGGGGAGCGAACAGGATTAGATACCCTGGTAGTCCACGCCGTAAACGGTGGATGCTGGATGTGGGGCCCTTTTTCCGGGTCCTGTGTCGGAGCTAACGCGTTAAGCATCCCGCCTGGGGAGTACGGCCGCAAGGCTAAAACTCAAAGAAATTGACGGGGGCCCGCACAAGCGGCGGAGCATGCGGATTAATTCGATGCAACGCGAAGAACCTTACCTGGGCTTGACATGTGCCGGACGCCCGCGGAGACGCGGGTTCCCTTCGGGGCCGGTTCACAGGTGGTGCATGGTCGTCGTCAGCTCGTGTCGTGAGATGTTGGGTTAAGTCCCGCAACGAGCGCAACCCTCGCCGCGTGTTGCCAGCGGGTCATGCCGGGAACTCACGTGGGACCGCCGGGGTTAACTCGGAGGAAGGTGGGGATGACGTCAGATCATCATGCCCCTTACGTCCAGGGCTTCACGCATGCTACAATGGCCGGTACAACGGGGTGCGACACGGTGACGTGGGGCGGATCCCTGAAAACCGGTCTCAGTTCGGATCGCAGTCTGCAACTCGACTGCGTGAAGGTGGAGTCGCTA-GTAATCGCGGATCA

**>BPSE BSM8-1**

TGTGAAAGTCCATCGCTTAACGGTGGATCCGCGCCGGGTACGGGCGGGCTTGAGTGCGGTAGGGGAGACTGGAATTCCCGGTGTAACGGTGGAATGTGTAGATATCGGGAAGAACACCAATGGCGAAGGCAGGTCTCTGGGCCGTTACTGACGCTGAGGAGCGAAAGCGTGGGGAGCGAACAGGATTAGATACCCTGGTAGTCCACGCCGTAAACGGTGGATGCTGGATGTGGGGCCCTTTTTCCGGGTCCTGTGTCGGAGCTAACGCGTTAAGCATCCCGCCTGGGGAGTACGGCCGCAAGGCTAAAACTCAAAGAAATTGACGGGGGCCCGCACAAGCGGCGGAGCATGCGGATTAATTCGATGCAACGCGAAGAACCTTACCTGGGCTTGACATGTGCCGGACGCCCGCGGAGACGCGGGTTCCCTTCGGGGCCGGTTCACAGGTGGTGCATGGTCGTCGTCAGCTCGTGTCGTGAGATGTTGGGTTAAGTCCCGCAACGAGCGCAACCCTCGCCGCGTGTTGCCAGCGGGTCATGCCGGGAACTCACGTGGGACCGCCGGGGTTAACTCGGAGGAAGGTGGGGATGACGTCAGATCATCATGCCCCTTACGTCCAGGGCTTCACGCATGCTACAATGGCCGGTACAACGGGGTGCGACACGGTGACGTGGGGCGGATCCCTGAAAACCGGTCTCAGTTCGGATCGCAGTCTGCAACTCGACTGCGTGAAGGTGGAGTCGCTA-GTAATCGCGGATCA
